# Recurrence of Pheochromocytoma With Metastases After Resection of Primary Tumor

**DOI:** 10.7759/cureus.8328

**Published:** 2020-05-28

**Authors:** Sharini Venugopal, Mamta Chhabria, Michael Quartuccio

**Affiliations:** 1 Internal Medicine, Rochester General Hospital, Rochester, USA; 2 Endocrinology, Diabetes and Metabolism, Rochester General Hospital, Rochester, USA; 3 Endocrinology, Diabetes and Metabolism, Johns Hopkins Hospital, Baltimore, USA

**Keywords:** size, pheochromocytoma, recurrence, benign, mutation, malignant

## Abstract

Pheochromocytomas and paragangliomas are rare tumors that arise from the chromaffin cells of the adrenal medulla or sympathetic paravertebral ganglia, respectively. Long-term surveillance is recommended regardless of the thoroughness of surgical resection. Here, we present a patient who was diagnosed with pheochromocytoma who underwent right adrenalectomy and was lost to follow up. She presented 15 years later with recurrence and was found to have multiple metastases. Subsequent genetic testing was also negative.

## Introduction

The annual incidence of these tumors is 0.8 per 100,000 person years [1]. Malignancy is currently defined as the presence of metastasis at nonchromaffin sites. While several features are correlated with a higher risk of recurrence or malignancy, no predictive models exist [2]. Clinical and pathological features also do not provide much information for estimating the risk of recurrence [3]. Surgical resection is not necessarily curative and these tumors can recur even decades after the original surgery. Therefore, long-term surveillance is crucial for these patients.

## Case presentation

**Figure 1:**
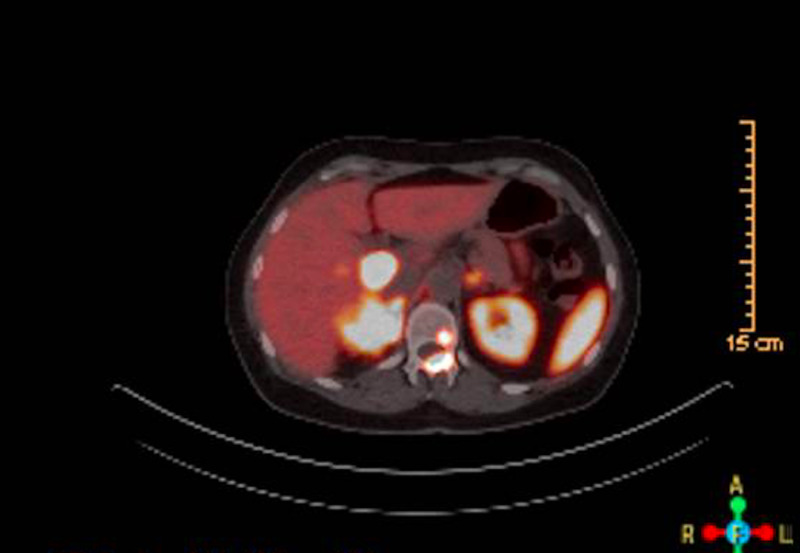
Right-sided adrenal mass lighting up on PET-dotatate scan. PET, positron emission tomography

This is a 55-year-old female who initially presented to another clinic with occasional palpitations and newly diagnosed hypertension, 15 years before her current presentation. At that time, she was also found to have elevated fasting glucose of 123 mg/dL during her initial prevention. Her blood pressure was difficult to control with systolic blood pressure in the range of 160s and therefore a secondary workup was pursued. A renal ultrasound excluded renal artery stenosis, but revealed a right-sided 5.1 cm x 4.1 cm x 5.8 cm adrenal mass. Lab work revealed markedly elevated urinary nor-metanephrines of 12522 nmol/d (normal range is 273-3548 nmol/d) and she was diagnosed with pheochromocytoma. After pre-treatment with doxazosin, she underwent laparoscopic right adrenalectomy within one month. The surgical pathology showed a 6.5 cm pheochromocytoma with focal infiltration at the periphery, but clear margins. The proliferative index was 6% based on Ki-67/MIB-1 staining. Synaptophysin immunostaining was diffusely positive consistent with the diagnosis. Genetic testing was not performed at that time and no post-surgical surveillance was performed as she was lost to follow up. She was asymptomatic for 15 years before presenting to her primary care physician, again with symptoms of palpitations and dizziness. Given her past history of pheochromocytoma, urine 24 hours nor-metanephrines were ordered and returned markedly elevated at 4323 mcg/24 hours (normal is 128-484 mcg/24 hours). A CT abdomen showed a 4.3 cm x 2.0 cm right adrenal mass, small caudate liver lesions, scattered aorto-caval nodes, and a lytic/sclerotic lesion on the first lumbar spine, all avid on Gallium-Dotatate Positron Emission Tomography, consistent with recurrent, metastatic pheochromocytoma as seen in Figure 1. 

Genetic testing did not reveal any common mutations associated with hereditary pheochromocytoma such as Myc-associated factor X (MAX), RET pro-oncogene, succinate dehydrogenate complex genes (SDHAF2, SDHB, SDHC, SDHD), transmembrane protein 127 (TMEM127), and Von Hippel-Lindau (VHL) tumor suppressor gene. She is currently being considered for medical therapy with Iobenguane Iodine 131 (Azedra).

## Discussion

Pheochromocytomas are rare tumors that are typically amenable to resection, but carry a recurrence rate of approximately 6.5%-16.5%. The most important fact to keep in mind is that surgical resection does not always result in cure, even in benign tumors. A retrospective, single institution study of 135 patients undergoing adrenalectomy for pheochromocytoma and followed for a 14-year period showed that large tumor size (>5 cm) was an independent risk factor for recurrence [4].

A small study of 192 patients showed that age, familial disease, tumor site (right-sided and extra-adrenal tumors), and size were independent predictors of recurrence. In the same study, pheochromocytoma recurred in 29 patients, out of which only 15 cases were malignant [5]. Timing of recurrence is also extremely variable, with some recurrences of metastatic disease having been discovered as long as 53 years after the initial surgery [6].

No single factor has been shown to predict the recurrence of these neoplasms, including pathological, clinical, or biochemical features [2]. Certain genetic mutations, such as SDHB, confer a higher risk for malignancy and recurrence, but testing is expensive and does not predict all cases, such as ours [7]. Therefore, long-term surveillance with biochemical tests and/or imaging modalities is needed even after surgical resection [8]. In addition, larger studies are required to investigate specific predictive markers for recurrence and to determine the appropriate duration of surveillance for these patients. 

## Conclusions

Pheochromocytomas are rare tumors and they carry the risk of recurrence rate even after complete resection. In our case, the side and size of the tumor could have predicted a higher risk for recurrence. However, the patient did not test for any genetic mutations that confer a higher risk, especially the SDHB mutation for which she tested negative. In addition, the patient had presented with the tumor that was confined to the adrenal gland that usually carries low risk from previous small studies. Because of the lack of accurate predictors for recurrence, current guidelines recommend lifetime annual biochemical screening for these patients after surgery. 
